# Utilizing 3D Printing Technology to Create Prosthetic Irises: Proof of Concept and Workflow

**DOI:** 10.3390/bioengineering10111287

**Published:** 2023-11-06

**Authors:** Alisa J. Prager, Nathaniel Henning, Lauren Burns, Abhijit Ramaprasad, Surendra Basti, Monica M. Laronda

**Affiliations:** 1 Department of Ophthalmology, Northwestern University Feinberg School of Medicine, Chicago, IL 60611, USAabhijit181@gmail.com (A.R.); sbasti@northwestern.edu (S.B.); 2 Stanley Manne Children’s Research Institute, Ann and Robert H. Lurie Children’s Hospital of Chicago, Chicago, IL 60611, USA; nathaniel.henning@northwestern.edu (N.H.); lauren_burns@alumni.brown.edu (L.B.); 3 Division of Endocrinology, Department of Pediatrics, Northwestern University Feinberg School of Medicine, Chicago, IL 60611, USA

**Keywords:** 3D printing, aniridia, artificial iris, iris prosthesis

## Abstract

Purpose: There are currently limited treatment options for aniridia. In this context, 3D printed iris implants may provide a cost-effective, cosmetically acceptable alternative for patients with aniridia. The purpose of this study was to develop a proof-of-concept workflow for manufacturing 3D printed iris implants using a silicone ink palette that aesthetically matches iris shades, identified in slit lamp images. Methods: Slit lamp iris photos from 11 healthy volunteers (3 green; 4 blue; 4 brown) were processed using k-means binning analyses to identify two or three prominent colors each. Candidate silicone inks were created by precisely combining pigments. A crowdsourcing survey software was used to determine color matches between the silicone ink swatches and three prominent iris color swatches in 2 qualifying and 11 experimental workflows. Results: In total, 54 candidate silicone inks (20 brown; 16 green; 18 blue) were developed and analyzed. Survey answers from 29 individuals that had passed the qualifying workflow were invited to identify “best matches” between the prominent iris colors and the silicone inks. From this color-match data, brown, blue, and green prototype artificial irises were printed with the silicone ink that aesthetically matched the three prominent colors. The iris was printed using a simplified three-layer five-branch starburst design at scale (12.8 mm base disc, with 3.5 mm pupil). Conclusions: This proof-of-concept workflow produced color-matched silicone prosthetic irises at scale from a panel of silicone inks using prominent iris colors extracted from slit lamp images. Future work will include printing a more intricate iris crypt design and testing for biocompatibility.

## 1. Introduction

The iris has two important roles. First, it provides eye color, which significantly contributes to facial appearance [1,2]. Second, the pupillary opening in the iris regulates light transmitted to the retina, increases the depth of focus during accommodation, and is the critical gateway determining the quality and quantity of vision. Patients with reduced iris function can experience decreased quality of life due to reduced cosmesis, increased light sensitivity, glare, blurry vision, and even loss of sight [3]. Aniridia, which is a partial or complete absence of the iris, affects many patients and can be congenital or acquired. Congenital iris defects include coloboma, albinism, Reiger’s syndrome, and other rare genetic anomalies. Acquired iris defects can result from trauma, excision of iris tumors, iatrogenic injury during intraocular surgery, and diseases such as uveitis and iridocorneal endothelial syndrome.

Currently, there are limited treatment options for aniridia. Conservative management options include sunglasses, occlusive devices, and tinted contact lenses [4]. Surgical options include iris reconstruction, intrastromal corneal tattooing, and artificial iris implants [5]. Corneal tattoos and contact lenses create opacity in the corneal plane, which is anterior to the pupil, resulting in a reduction in peripheral vision and light scatter. While iris suturing may be adequate for small iris defects, there may not be enough iris tissue to repair larger defects. Furthermore, many patients with large iris defects are not satisfied with conservative management that may only partially alleviate their symptoms. Therefore, there have been efforts to develop surgically implanted iris prostheses that are safe, effective, and satisfactory for patients.

One of the challenges of treating aniridia is that there are currently limited options for commercially available, cosmetically acceptable prosthetic iris implants. Since a majority of the cases of aniridia are unilateral, one requirement for a desirable prosthetic iris is to closely match the color of the iris of the other eye. In addition, the use of flexible, biocompatible materials such as silicone would reduce the incision size for implantation and minimize adverse events [6]. Black diaphragm intraocular lenses (Morcher GmBH, Stuttgart, Germany) have a central opening with a black periphery simulating a normal iris and may be useful for patients with aphakia and aniridia [7]. However, these lenses are not cosmetically acceptable for lighter-colored irises, are rigid, require large 150-to-180-degree sclerocorneal surgical incisions, and have been associated with a high incidence of serious complications that limit their wide-spread use [8,9]. Modified capsular tension rings with black occluder paddles have also been utilized, and while these require smaller surgical incisions during implantation, they have a similar limitation of unacceptable cosmesis as black diaphragm intraocular lenses [10]. Reper devices (distributed by Ophtec BV, Groningen, The Netherlands) are made of foldable hydrophobic acrylic material with different color options available; however, the iris color and pattern are not customizable [11]. One of the challenges of iris implants is the accurate reproduction of a human iris with pigmented, layered texture with crypts and folds. Currently, the only FDA-approved prosthetic iris, the CustomFlex Artificial Iris by HumanOptics (Erlangden, Germany), is a custom-made pliable silicone prosthesis that is hand-painted in Germany based on the appearance of the patient’s other eye [12,13,14]. The clinical trial for this implant met safety and efficacy endpoints and demonstrated a significant reduction in light sensitivity and glare as well as an improvement in cosmesis and quality of life measures [15]. However, the limitations of this custom-made device include its high cost and three- to four-month production time. Furthermore, the CustomFlex Artificial Iris may not be covered by some insurance carriers, limiting access to patients that need it.

Recently, 3D printing has been utilized in healthcare to provide solutions for clinical challenges. With the right materials and appropriate printing parameters, 3D printing technology can be used to generate biocompatible, cost-efficient, and customizable ocular therapies and implants for patients. The benefit of 3D printing is increased production speed, reproducibility, and ease of printing adjustments, which allow for mass production as well as customizability. Thus far, 3D printing has been utilized for multiple ophthalmic applications, including the production of model eyes for diagnostics [16], surgical education [17], surgical implants [18], surgical instrumentation and devices [19], and ocular prostheses [20,21]. Of particular interest is the report of the production of a realistic 3D printed ocular prosthesis by Groot et al. in 2021. They used computer-aided designs to 3D print an ocular prosthesis with a full colored, textured iris and sclera in a single print job [22]. While this is promising, the material and inks used for this ocular prosthesis are not biocompatible for intraocular use.

To the best of our knowledge, there have been no prior studies on the application of 3D printing for the creation of a stand-alone prosthetic iris implant (without the ocular prosthesis). This study was initiated with the aim of developing a proof-of-concept workflow for a cost-effective silicone iris prosthesis. We sought to create a prototype that aesthetically matches the patient’s other eye and utilizes slit lamp photography, digital and survey-based color-matching, and 3D printing to achieve this goal.

## 2. Materials and Methods

### 2.1. Reference Iris Imaging and Analysis

Reference iris photos were collected from 11 technical staff volunteers from the Northwestern Medicine ophthalmology department. Volunteers were chosen to include the three predominant iris colors in the human population (blue, green, and brown). Slit lamp photos of one eye of each volunteer were taken using a Haag-Streit BX-900 Slit Lamp (Metall Zug, Zug, Switzerland) with an attached Canon EOS 7D DSLR camera (Ōta, Tokyo, Japan). Of the 11 iris images, 4 were subjectively categorized as containing a majority of brown hues, 4 as blue, and 3 as green. The images were white-balanced using an open-source ImageJ script (Bindokas V, 2006, Univ. of Chicago, IL, USA; modified by Mascalchi P, 2014, Univ. of Cambridge, Cambridge, UK) [23,24]. The sclera, pupil, and external areas were cropped from the photos to isolate the iris. Using a custom R script (Supplementary Material), the three most prominent color pixels in each iris photo were determined using k-means binning [25] by least within-group variation. The third prominent iris color within one brown iris (designated “F”) was a dark grey and not within our inclusion criteria. Therefore, this iris analysis only resulted in 2 instead of 3 prominent colors, and 32 iris colors from the 11 volunteers were used in our survey analysis (as described in “Color matching survey”).

### 2.2. Silicone Pigment Mixing

Seven different silicone pigments, green, yellow, white, black, brown, “blood” (red), and blue (Silc Pig silicone pigments, Smooth-On Inc., Macungie, PA, USA) were mixed into GE Advanced Silicone II (Momentive Performance Materials Inc., Huntersville, NC, USA, model #GE5040) in precise combinations to make 72 shades of printable silicone inks. Because some silicone pigment tones were significantly different from the natural iris hues, the silicone pigments were down-selected to 54 for survey analysis (as described in “Color matching survey”). Candidate inks were photographed using the same slit lamp camera apparatus and settings as the iris photos. The images were white-balanced using the same ImageJ script, and the central area of the photos was cropped. Using the R k-means script, a single prominent color was determined for each candidate ink (Supplementary Figure S1).

### 2.3. Color-Matching Survey

To evaluate subjective color matches between candidate inks and iris colors, volunteers were recruited through distribution of a link that was open to the public on Zooniverse (Zooniverse.org accessed on 24 October 2023). Zooniverse is a free online platform that supports crowd-sourced research. This study was reviewed and approved by the Ann & Robert H. Lurie Children’s Hospital of Chicago Institutional Review Board (IRB 2022-4892). The Zooniverse survey consisted of two qualifying workflows, where a single brown, blue, or green color swatch was presented, and participants indicated whether it was brown, blue, or green. If the participant answered those questions correctly, they were then presented with 11 iris (experimental) workflows, where each iris workflow represented the colors of one volunteer iris photo. In the iris workflows, color swatches of each of the two to three prominent colors within the iris (labeled with letters) were compared with color swatches for each candidate ink for the iris’s general color category (brown, blue, or green, labeled with numbers). Five hundred and eighty pairs of silicone ink to prominent iris color were created and the participant chose “yes” or “no” in response to the question “Do these colors match?” Twenty-nine participants matched any of the five hundred and eighty silicone ink to iris color combinations. To ensure that all combinations were reviewed sufficiently, color combinations that had received responses from 10 individuals were no longer available to answer on Zooniverse. Responses were totaled and the silicone ink hues were assessed for matches (i.e., “yes” responses) for a given iris color.

### 2.4. 3D Printing

A simple three-color iris prosthetic computer-aided design was developed using Autodesk Fusion 360 (San Francisco, CA, USA). The design was sliced into three layers: (1) a 12.8 mm disk base layer with a 3.5 mm central opening for the pupil, (2) a starburst layer representing crypts, and (3) a superficial layer using the same starburst pattern at a 30-degree offset. The ink colors with the greatest number of participants who answered affirmatively to the iris color match were chosen for and used in the 3D printed design. Each layer of the three-layer design was assigned a different ink color to represent the three prominent colors of the iris. In cases where participants were unable to rate a single-color comparison as most similar, a color was subjectively chosen by project leads from the color options with the highest affirmative scores given by survey participants. The design was printed using an EnvisionTEC Bioblotter (Manufacturer Series, ETEC, Gardena, CA, USA) clear 27-gauge (0.2 mm) SmoothFlow tips (#7005008, Nordson EFD, Nordson Corporation, East Providence, RI, USA). Because the only FDA-approved prosthetic iris that meets the biocompatibility requirements for this surgically implanted device is made of silicone (FDA Premarket Approval Number P170039), we chose to use silicone to develop the proof-of-concept workflow.

## 3. Results

### 3.1. Manufacturing Silicone Pigments Based on Iris Photographs

**Figure 1:**
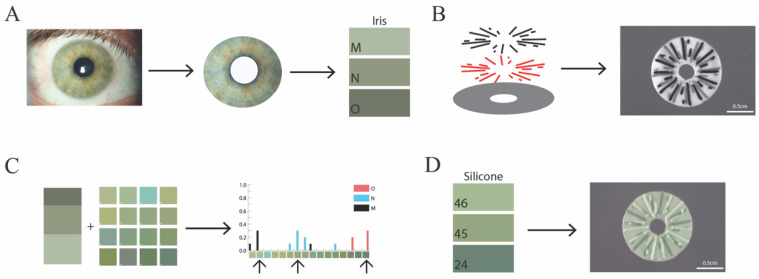
Schematic of workflow for developing a green 3D printed prosthetic iris. (**A**) Slit lamp image of green iris was processed to identify the three most prominent colors (M, N, O). (**B**) Simplified iris design with solid base and two five-point starburst pattern slices offset by 30 degrees. This design was printed in white, grey, and black silicone at scale. (**C**) Results from the survey comparing 3 prominent green colors of the iris with 16 green silicone ink hues identified the 3 inks that best matched the iris colors. (**D**) The silicone inks of the 3 hues were used to print a silicone iris in a 3-layer pattern. Scale bars, 0.5 mm.

**Figure 2:**
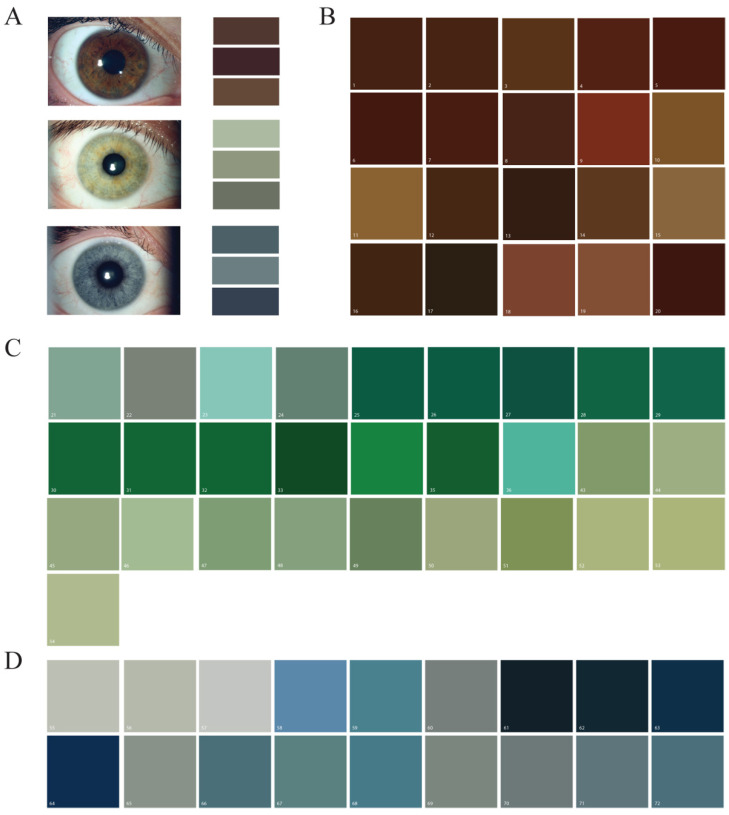
Prominent iris colors and silicone ink hues. (**A**) Representation of brown, green, and blue irises and the three prominent colors identified. (**B**) Brown, (**C**) green, and (**D**) blue silicone iris ink hues imaged using slit lamp photography.

Eleven individuals volunteered to have their iris imaged using a slit lamp camera. The dominant iris colors were extracted from slit lamp photographs of the irises using processing and custom R-scripts (Figure 1). The three most prominent colors were identified for 11 iris images (Figure 2A) to establish the intended three colors for printing. Concentrated silicone pigment was added at volumes that maintained printability for extrusion printing. In total, 72 hues (20 shades of brown, 34 greens, and 18 blues) were generated by combining two or three pigment colors into a clear silicone base. Fifty-four of these silicone inks were down-selected for additional analysis and imaged using the same slit lamp camera used for the volunteer iris images to ensure consistency in lighting and hue of the color represented in the image (Figure 2B–D).

### 3.2. Silicone Color-Matching Survey Results

**Figure 3:**
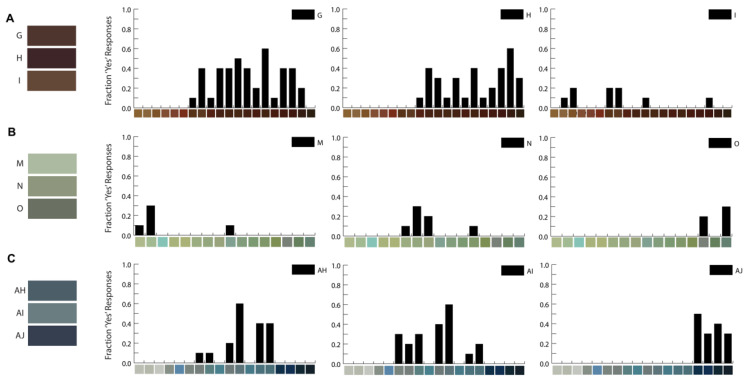
Example results from color-matching survey. (**A**) The brown iris colors (G, H, I), (**B**) green iris colors (M, N, O), or (**C**) blue iris colors (AH, AI, AJ) were present next to the corresponding silicone ink hues along the x axis. The fraction of “yes” respondents to the question “Do these colors match?” are presented.

An online survey was designed and implemented to survey individual perspectives on which silicone ink hues matched the two or three prominent colors identified within volunteer irises. Participants were asked to respond to ink color swatches adjacent to the iris color swatches based on their immediate initial impressions of likeness. A total of 580 silicone-ink-to-iris-color combinations were surveyed to determine which silicone ink hues were aesthetically similar to the prominent colors identified in the volunteer irises (Supplementary Figure S1). It was possible to respond “yes” to more than one silicone ink hue per iris color. The percentage of individuals who matched a silicone ink hue to the iris color presented (i.e., answered “yes”) ranged from 0 to 90% for each iris-color-to-silicone-ink hue pair (Figure 3, Supplementary Figure S1). Of the 32 iris colors used in the surveys, 13 were matched to a specific silicone ink hue by at least 50% of the respondents, and 19 were matched by at least 40% of respondents. Many (8/12) brown iris colors were matched to multiple ink hues, with over 40% of respondents answering “yes”. Some iris colors were not matched with a silicone ink hue. The brown iris colors were matched with the most silicone ink hues, followed by blues, then greens. The silicone ink hues with the highest matches for a given iris color were chosen as the three candidate silicone inks to represent the prominent colors in a 3D printed prosthetic iris.

### 3.3. 3D Printing of Colored Irises

**Figure 4:**
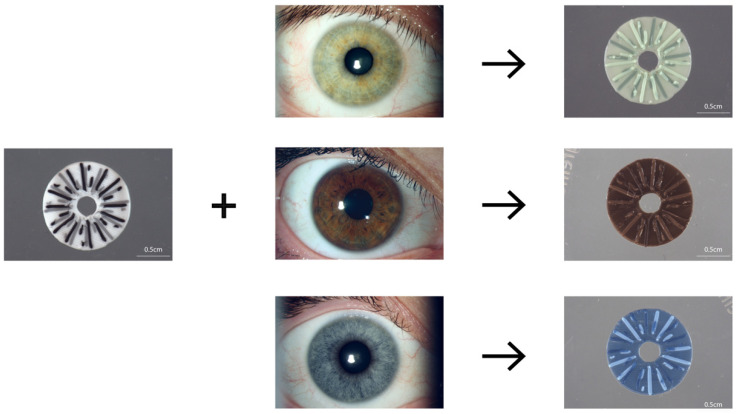
Simplified silicone prosthetic iris in color-matched hues, 3D printed to scale. Representation of brown, green, and blue prosthetic irises from silicone ink hues matched in the survey. The silicone ink hues are each printed as either the base or one of the starburst layers. Scale bars, 0.5 mm.

A simplified computer-aided iris design was produced to mimic crypt details while maintaining practical silicone 3D printability and resolution at a transplantable scale. It was determined that 35 μL or less of colored pigment could be added to clear silicone without disrupting the printability of the ink. The simplified iris design was a 12.8 mm base disc with a centralized 3.5 mm hole, representing the pupil. The two layers of a 5-branch starburst, one offset by 30 degrees, was used to represent crypt details (Figure 1 schematic). Silicone ink hues were chosen based on the survey data detailed in “Silicone color matching survey results” for each of the three prominent volunteer iris colors. The ink colors were alternated as the silicone ink chosen for the base or two starburst layers (Figure 4). A subjective best-match to the reference photo of the iris for green-, brown-, and blue-colored irises provides a proof of concept for this manufacturing workflow.

## 4. Discussion

The aim of this study was to develop a proof-of-concept workflow for creating a cost-effective 3D printed silicone prosthetic iris. We used slit lamp photography of irises of three primary colors and digital and survey-based color matching to arrive at the hue and intensity that best represented irises of these three color palettes. The printed prototype must be of an appropriate size, biocompatible, and flexible for transplantation. Silicone was chosen because it is the material used in the only existing FDA-approved transplantable iris device and is printable by extrusion-based printing. Achieving a prosthesis that aesthetically matches the color of the other eye was also an important step for creating such a prosthesis, which aims to improve the quality of life for aniridia patients. While 3D printing technologies have been utilized in the production of ocular prostheses with an iris design that is either hand-painted [26], printed utilizing a sublimation technique [27,28], or produced in a single print job utilizing non-silicone material [22], these results advance the field by utilizing 3D printing with silicone inks to generate an aesthetically color-matched iris. One of the challenges of 3D printing an iris is the development of appropriate color inks that resemble the natural eye and printing structural details to create the natural texture of the iris with crypts and folds. Additional drawbacks of currently available prosthetics include the time and costs to generate colored irises. Many of these challenges have been circumvented with the protocol presented herein.

In the United States, surgical options to treat aniridia have included the use of artificial iris–intraocular lens prosthesis (e.g., black diaphragm intraocular lenses), a modified capsular tension-ring-based artificial iris, and customized artificial iris implants (e.g., HumanOptics silicone prosthetic iris) [29]. A black diaphragm intraocular lens (IOL) may not be cosmetically acceptable for lighter-colored irises, and while glare symptoms improve significantly, resulting in a six-line improvement in Snellen acuity and approximately fifty percent decrease in patient reported glare symptoms in bright and dim light, the prosthesis has been associated with a high incidence of serious complications for patients [8,9]. These post-implantation complications typically take place within one year of surgery and can include eccentric positioning, glaucoma, corneal decompensation, limbal stem cell failure, and uveitis with ocular hypertension [8]. In contrast to the options above, the HumanOptics prosthetic iris provides an exceptional color and pattern match to the iris of the other eye. Patients followed for a year with this iris implant showed a statistically significant decrease in both day and nighttime glare as well as light sensitivity. Patient outcomes surpassed all key safety endpoints [10]. However, the process and time to procure this prosthetic iris are significant, because it is hand-painted in Germany. The whole process is also expensive, making it a surgical intervention often denied coverage by insurance carriers. Due to the above considerations, there is room for a device that obviates the downsides of currently available options. Our study demonstrates the potential for 3D printed silicone iris prosthesis to avert the downsides of those options and provide a cosmetically, visually acceptable, and potentially cost-effective solution.

The role of 3D printing in medicine is constantly evolving and expanding. Today, 3D printing, or additive manufacturing, can be carried out through a variety of processes, with material being added layer by layer. In medicine, images are typically acquired with cameras or CT or MRI machines and then processed to generate a 3D model utilizing CAD software. Then, the 3D model is printed using customizable materials and techniques. Traditionally, 3D printing has used conventional materials including polymers. More recently, 3D bioprinting has utilized natural and synthetic biomaterials, including cells, to create complex organs [30].

In addition, 3D printing technology has been applied to the fabrication of both ocular and intraocular ophthalmic devices, anatomical models, and potential therapies [31,32,33]. As mentioned previously, 3D printing has been applied to the creation of patient-specific ocular prostheses [27], orbital implants for orbital fractures, and anophthalmic socket contractors. For intraocular implants and therapies, milestones include the synthesis of the first intraocular pupil expansion device in 2015 and the first 3D printed cornea in 2018 [32,34,35]. Patient-specific 3D printed intraocular lenses [36] have also been reported and hold the promise of correcting patient-specific refractive errors and unusual anatomic features. However, several technological challenges have to be overcome before this achieves mainstream use. Lastly, 3D bioprinting is also being explored for drug delivery systems and the regeneration of retina [37] and cornea [27,28,29,30,33], which aims to help address the shortage of donor corneas. The customization of 3D printing technology would allow the production of glaucoma implants such as drug-eluting implants, drug-loaded punctal plugs, and minimally invasive glaucoma devices using selected biomaterials customized to each patient.

One of the greatest challenges in 3D printing of an artificial iris is to make an accurate representation of the remaining eye’s iris in terms of style, color, and scale. Firstly, because the complex crypts within the iris do not translate well to printed silicone ink using this extrusion-based method at the scale of an iris diameter, a simplified print design was used here in the proof-of-concept workflow. Secondly, we chose to develop an array of brown, blue, and green hues that were within our silicone, color-mixing abilities and did not disrupt the printability of the silicone by diluting it with liquid-based color. Finally, we surveyed for silicone ink hues that matched the hues within the iris image to achieve aesthetically matched colors that could be printed. While the survey for matching colors had a small sample size, the distributions of the responses for each color revealed that individual perceptions of whether a color matches another color is not uniform. Additionally, several iris colors were matched to the same silicone ink colors. From these observations, silicone inks of a select number of hues may be required to support an aesthetically matched prosthetic iris, especially for darker colors. Such an approach will also have lower costs of testing, manufacturing, and storing. Future work to use digital or automated comparisons of prominent iris colors that can be translated to silicone ink hue formulas would improve the efficiency of the color match process and identify which silicone inks could be stocked for prosthetic iris printing. The current project used a simplified print. A series of more complex crypt structures could be integrated into the manufacturing process without adding additional printing time. Finally, manipulations of the silicone ink formulae could improve printability and reduce the printed strut thickness, enabling finer details within the crypts.

While showing promise, this approach is a beginning, and many additional steps are required to prove its efficacy, safety, and validity. While silicone is currently used as a prosthesis, these 3D printed prostheses generated with colored silicone have yet to undergo biocompatibility testing. Preclinical studies will be necessary to assess intraocular color stability, biocompatibility, and material longevity. Despite these limitations, our proof-of-concept workflow is an important step in the development of 3D printed iris implants. The protocol used has the potential to enable the creation and wide-spread application of these implants, with the distinct possibility to lower costs, reduce production times, and ultimately provide much easier and improved access to prosthesis for patients with aniridia.

## 5. Conclusions and Future Directions

The application of 3D printing in ophthalmology has led to the development of new therapeutic options for different types of ophthalmic conditions. The use of this technology for the production of iris implants is a promising next step in improving the quality of life for patients with aniridia. This proof-of-concept workflow demonstrates the potential use of color-matching surveys and 3D printing to produce an aesthetically matched silicone iris prosthesis. The potential advantages over current options include a significant cost reduction, reproducibility, and efficiency, as well as customizability and cosmetic acceptability.

Before these implants can be used in humans, however, additional preclinical testing will be necessary to assess its safety and feasibility. Future directions include developing appropriate intraocular delivery methods and surgical techniques, as well as testing for biocompatibility in preclinical animal models. Additional clinical testing is necessary to critically evaluate the potential role of these implants for clinical use.

## Data Availability

The data presented in this study are available on request from the corresponding author.

## Supplementary Materials

The following supporting information can be downloaded at: https://www.mdpi.com/article/10.3390/bioengineering10111287/s1.
