# Supplementary figures and images for: Utilizing 3D Printing Technology to Create Prosthetic Irises: Proof of Concept and Workflow
Source: Bioengineering (Basel). 2023 Nov 6;10(11):1287. doi: 10.3390/bioengineering10111287 (PMC10669136; doi:10.3390/bioengineering10111287)

Supplementary Figure S1

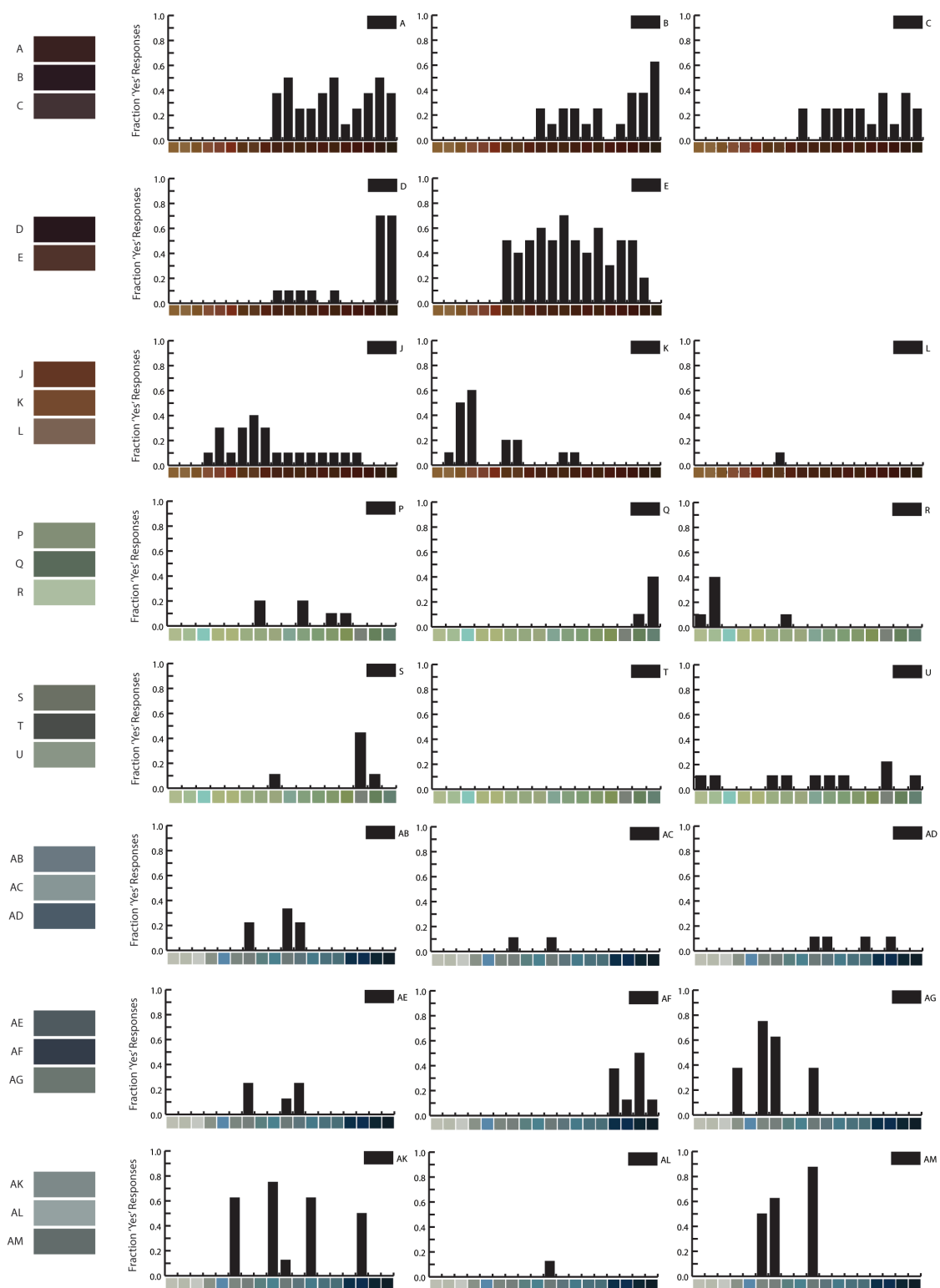

Supplement: Supplementary file 1 [file bioengineering-10-01287-s001.zip › bioengineering-2604808-supplementary.pdf]
